# Risk scoring model for lung adenocarcinoma based on PD-L1 related signature reveals prognostic predictability and correlation with tumor immune microenvironment genes was constructed

**DOI:** 10.3389/fimmu.2025.1601982

**Published:** 2025-06-11

**Authors:** Meng Li-Fei, Si-Meng Ren, Jun Wang, Wei-Jun Zhao, Jian Chen, Wen-Tao Hu

**Affiliations:** ^1^ Department of Thoracic Surgery, The First Affiliated Hospital of Ningbo University, Ningbo, China; ^2^ Department of Psychology, College of Liberal Arts, Wenzhou-Kean University, Wenzhou, China

**Keywords:** lung adenocarcinoma, programmed death ligand-1, bioinformatics, survival analysis, machine learning

## Abstract

**Background:**

Immunotherapy has recently become a hot topic in the field of oncology, with PD-L1 playing a crucial role in this area. However, the research on PD-L1 correlation prediction models is not fully understood. The aim of our study was to investigate the role of PD-L1-related genes in lung adenocarcinoma immunity.

**Methods:**

The mRNA and clinical data were obtained from the Cancer Genome Atlas database. DESeq2, Glmnet, forestplot, clusterProfiler and enrichplot were used to analyze the mRNA and clinical data. Western blotting and real-time qRT-PCR were used to confirm the GPR115, MF12, GREB1L, SPRR1B and LIPK mRNA and protein expression.

**Results:**

Firstly, 562 cases of TCGA lung adenocarcinoma, including 503 of tumor tissue and 59 of normal tissue were collected. The dataset was analyzed using the DESeq2 package of R. 1,251 high- and 285 low-expression genes were obtained. The tumor samples were divided into CD274-high and CD274-low expression samples and 873 genes were up-regulated and 1,010 genes were down regulated between CD274-high and CD274-low samples. Subsequently, the intersection of 1,251 and 873 was taken to obtain 110 genes that were both highly expressed genes in tumors and CD274 high-expression samples. Survival analysis of 110 genes yielded 5 meaningful genes including GPR115, MF12, GREB1L, SPRR1B, and LIPK (p < 0.001). These five genes were used to construct PD-L1 risk predictors. Cytokine-cytokine receptor interaction and IL-17 signaling pathway were involved in the regulation of this risk model factors to lung adenocarcinoma. The level of effector memory CD4 T cells and Type 2 T helper cells were correlated with the risk model factor. Importantly, the PD-L1 risk prediction model could effectively predict the prognosis of patients.

**Conclusion:**

The construction of PD-L1 risk model was of great significance for the treatment of lung adenocarcinoma.

## 1 Introduction

As the most common histological subtype of non-small cell lung cancer (NSCLC), lung adenocarcinoma has a high incidence and mortality rate that seriously threatens human health (1, 2). Although significant progress has been made in targeted therapy and immunotherapy in recent years, tumor heterogeneity, drug resistance, metastasis and recurrence remain major challenges in clinical management (3–5). It is of great significance to deeply analyze the molecular mechanism of the occurrence and development of lung adenocarcinoma, especially the key genes that drive tumor progression, for the development of novel biomarkers and precision treatment strategies. In recent years, several studies have suggested that genes such as SPRR1B, MFI2, LIPK, GREB1L, and GPR115 may play a potential role in lung adenocarcinoma (LUAD).

As a member of the transferrin family, the transmembrane protein MFI2 (Melanotransferrin, also known as CD228) has gradually become a research hotspot due to its potential role in tumor metabolic reprogramming and metastatic microenvironment regulation (6). In lung adenocarcinoma, abnormally elevated MFI2 expression was significantly associated with lymph node metastasis, late clinical stage, and shortened survival (7–9). Mechanistic studies suggest that MFI2 may enhance the migration and invasion ability of tumor cells through integrin-extracellular matrix interactions, or affect the drug resistance and immune escape of tumor cells by regulating iron-dependent metabolic pathways (such as lipid peroxidation). In addition, the potential association between MFI2 and immune microenvironment remodeling events such as tumor-associated macrophage (TAMs) polarization and PD-L1 expression is also worth further exploration (10–13). However, there is still a lack of systematic research on the functional mechanism, upstream regulatory network and feasibility of MFI2 as a therapeutic target in lung adenocarcinoma.

Growth regulation by estrogen in breast cancer 1-like (GREB1L) has become a research hotspot due to its potential function in developmental regulation and tumorigenesis (14, 15). GREB1L was initially found to be involved in embryonic organ formation and gonadal development, and subsequent studies suggest that it may affect cell proliferation and differentiation by regulating hormone receptor (e.g., estrogen receptor, androgen receptor) signaling or non-canonical pathways (14, 15). In the field of tumors, aberrant expression of GREB1L has been reported in breast, ovarian and prostate cancers, but its biological significance in lung adenocarcinoma is not clear (16–19). As an important regulator of triglyceride hydrolysis and fatty acid metabolism, the lipid metabolism-related enzyme LIPK (Lipase K) may disrupt lipid homeostasis, affecting the energy supply, membrane structure remodeling, and signal transduction of tumor cells, thereby driving the progression of lung adenocarcinoma (20–22). There is still a lack of systematic exploration regarding the specific targets of LIPK in lung adenocarcinoma, the mechanisms of epigenetic regulation, and its feasibility as a target for metabolic therapy.

Small Proline-Rich Protein 1B (SPRR1B), as a member of the epidermal differentiation-related protein family, has attracted much attention for its potential role in cell proliferation, migration, and Epithelial-Mesenchymal Transition (EMT) (23–25). EMT is a process by which epithelial cells transform into mesenchymal - like cells, endowing them with enhanced migratory and invasive properties (26, 27). Studies have shown that SPRR1B is abnormally high expressed in a variety of solid tumors (such as esophageal cancer, head and neck squamous cell carcinoma), and promotes tumor invasion and metastasis by activating signaling pathways such as EGFR/MAPK (23, 28–30). In lung adenocarcinoma, the expression of SPRR1B is significantly up-regulated and is closely related to the poor prognosis of patients. Further mechanistic studies have found that SPRR1B may enhance the migration ability and drug resistance of cancer cells by regulating cytoskeletal remodeling, matrix metalloproteinase (MMPs) secretion, and tumor microenvironment interaction (31, 32). However, the specific downstream targets of SPRR1B in lung adenocarcinoma, the epigenetic regulatory mechanism, and its association with the immune microenvironment are still not fully elucidated.

GPR115 (Adhesion G Protein-Coupled Receptor V1, ADGRV1), as a member of adhesion GPCRs, is involved in tissue development and homeostasis maintenance by mediating cell-matrix interaction and transmembrane signaling. In recent years, studies have suggested that GPR115 is abnormally expressed in a variety of malignant tumors (such as breast cancer and glioma), which may promote tumor invasion and drug resistance by activating MAPK/ERK or PI3K/AKT pathways (33–36). In lung adenocarcinoma, the expression of GPR115 was significantly up-regulated, and was significantly associated with TNM stage progression, distant metastasis, and shortened overall survival. Preliminary mechanistic studies suggest that GPR115 may enhance tumor cell adhesion and migration by regulating the integrin-FAK signaling axis, or promote tumor angiogenesis by mediating vascular endothelial growth factor (VEGF) secretion (37, 38). However, the mechanism of ligand-receptor interaction, epigenetic regulatory model, and pharmacological feasibility of GPR115 as a therapeutic target in lung adenocarcinoma still need to be elucidated.

The programmed death-1 (PD-1) pathway is a key mediator of local immunosuppression of the tumor microenvironment (TME) and also regulates T cell activation of tumor antigens and secondary lymph nodes (39). Blocking the PD-1 pathway by inhibiting PD-1 receptors on immune cells or PD-L1 ligands on tumors or immune cells can inhibit tumor growth and may lead to curability. PD-L1 antibodies, including Pembrolizumab, Nivolumab, Atezolizumab and Durvalumab, have been approved by the FDA for the clinical treatment of NSCLC (40). NSCLC still has a poor prognosis, and immunotherapy (IMT) has become part of the treatment of patients with no driver alterations (epidermal growth factor receptor (EGFR) or anaplastic lymphoma kinase (ALK)) (41). Guidelines from the ASCO and OH Joint Expert Group recommend pembrolizumab for non-squamous cell carcinoma (non-SCC) with high PD-L1 expression (Tumor Proportion Score [TPS]≥50%) (41). At present, immunotherapy has shown significant efficacy in patients with NSCLC with high expression of programmed death-ligand 1 (PD-L1) and high tumor mutational burden (42). Therefore, it is crucial to find predictive biomarkers of immunotherapy efficacy.

Immunotherapy has recently become a hot topic in the field of oncology. However, the research on PD-L1 correlation prediction models is not fully understood. The aim of our study was to investigate the role of PD-L1-related genes in lung adenocarcinoma immunity.

## 2 Materials and methods

### 2.1 Data acquisition and processing of lung adenocarcinoma

The Cancer Genome Atlas (TCGA) database, jointly established by the U.S. National Cancer Institute (NCI) and National Human Genome Research Institute (NHGRI), provides multi-omics data including transcriptomic expression profiles, genomic variation data, and clinical annotations. Clinical datasets were retrieved from the TCGA portal (http://portal.gdc.cancer.gov/), and 562 samples meeting inclusion criteria (complete clinical metadata) were retained for subsequent analyses. All datasets utilized in this study were derived from public repositories, thus exempting the requirement for ethics committee approval.

### 2.2 Screening of differentially expressed genes and enrichment analysis

Differentially expressed genes (DEGs) in lung adenocarcinoma were identified using the DESeq2 package in R (version 4.3.0), with thresholds set at an adjusted p-value (Benjamini-Hochberg false discovery rate [FDR]) < 0.05 and absolute log2-fold change (|log2FC|) > 1. Visualization of DEGs was performed using ggplot2 (volcano plots) and heatmaps (hierarchical clustering heatmaps). Subsequently, gene expression profiles were integrated with survival data (overall survival status and time). Prognostically significant genes were preliminarily screened through univariate Cox regression analysis (threshold: p < 0.05). Functional annotation of these prognosis-related genes was conducted using clusterProfiler and enrichplot packages, including: Gene Ontology (GO) enrichment analysis (biological processes, molecular functions, cellular components), Kyoto Encyclopedia of Genes and Genomes (KEGG) pathway analysis. Results were visualized via dot plots, enrichment maps, and circular dendrograms following best practices for omics data visualization.

### 2.3 Construction of protein-protein interaction network and correlation analysis

The GeneMANIA platform (http://genemania.org/), a widely used online tool for predicting protein-protein interactions and functional associations, was employed to construct a PPI network. The PPI network of these mitochondrial-related prognostic genes was subsequently constructed using the GeneMANIA database.

### 2.4 Construction of prognostic risk score model

Differentially expressed genes (DEGs) were integrated with LUAD patient survival data, and samples with incomplete clinical information were excluded. The Glmnet package in R was utilized to perform LASSO (Least Absolute Shrinkage and Selection Operator) regression, followed by Cox proportional hazards modeling for survival analysis. Forest plots were generated using the forestplot package. To mitigate overfitting in the prognostic model, genes identified through univariate Cox regression were subjected to LASSO regression via the Glmnet package, excluding genes with regression coefficients of zero. Subsequently, multivariate Cox regression analysis was performed to identify genes significantly associated with prognosis. A risk score was calculated for each patient based on the final gene set.


$$risk\;score=\sum_{i=1}^{n}Coef(i)\times m(i)$$

The final prognostic risk score model was constructed using the following parameters:

n: Total number of genes significantly associated with prognosis
Coef: Regression coefficient of each gene derived from multivariate Cox regression analysis
m: Expression level of each gene

### 2.5 Evaluation and validation of the prognostic risk model

A total of 503 LUAD samples were stratified into high and low risk groups based on the optimal cut-off value determined by receiver operating characteristic (ROC) curve analysis. The pheatmap package was utilized to generate: a survival status heatmap illustrated the distribution of high and low risk groups. An expression heatmap of prognosis-associated genes were used in the model. Survival curves were plotted using the survival package, and the independent prognostic value of the risk score was validated through Cox regression analysis. The predictive performance of the model was further evaluated by constructing ROC curves using an internal validation dataset.

### 2.6 Patient sample collection

Samples were collected from patients with NSCLC in the Department of Thoracic Surgery, the First Affiliated Hospital of Ningbo University. All patients provided written informed consent. NSCLC tissues and adjacent tissues were collected for research purposes. The samples were sectioned and stored frozen in liquid nitrogen at -80 °C. All procedures involving human participants were conducted in accordance with the 1964 Declaration of Helsinki and its subsequent amendments.

### 2.7 Western blotting

Proteins were extracted from tumor tissues and separated by polyacrylamide gel electrophoresis (Beyotime, Shanghai, China). The separated proteins were transferred to a polyvinylidene fluoride (PVDF) membrane (Invitrogen, USA) using an electro-transfer device (Servicebio, Wuhan, China), which was placed in an ice bath. Blocking was performed with 5% bovine serum albumin (BSA; Beyotime, Shanghai, China), and the membrane was placed on a shaker at a slow speed for 1 hour. The primary antibody was then added for incubation to allow specific binding, followed by incubation with horseradish peroxidase (HRP)-labeled secondary antibody to form an HRP-primary antibody conjugate (1:10000, Abbkine, China). The details of the primary antibodies are as follows: GREB1L (1:1000, Novus, USA), MFI2 (1:1000, CST, USA), SPRR1B (1:1000, Zen-bio, China), GPR115 (1:1000, Baijia, China), LIPK (1:1000, Novus, USA), and β-actin (1:1000, Proteintech, USA). Enhanced chemiluminescence (ECL; Millipore, USA) was used for signal development, which was stopped once clear bands appeared. The film was scanned, and the gray values of the target bands were analyzed using ImageJ software.

### 2.8 RNA isolation and real-time qRT-PCR

Total RNA was extracted from NSCLC cells using TRIzol reagent (Invitrogen, USA), and the RNA concentration was measured. qRT-PCR was performed using SYBR Green (Tiangen, China) on an ABI Illumina instrument (Foster, USA). The information of primers was listed in **Table 1**.

**Table 1:** The sequence of primer.

GREB1L	Forward primer	5’-ACCTCTGCCTCCCAGATGTC-3’
	Reverse primer	5’-CTTGTCTGAAACCAGGGGCA-3’
MFI2	Forward primer	5’-GGCACACAACCGTCTTGAC-3’
	Reverse primer	5’-GGGGCACAGCAGTTCATAGT-3’
SPRR1B	Forward primer	5’-ACCTCTGCCTCCCAGATGTC-3’
	Reverse primer	5’-CTTGTCTGAAACCAGGGGCA-3’
GPR115	Forward primer	5’-TGCCACGTGATGGTGAAGAT-3’
	Reverse primer	5’-TGGATCCTTCCAGTCTTGGG-3’
LIPK	Forward primer	5’-TCTCTTCACACCAGGACCAG-3’
	Reverse primer	5’-ACTATTGAAGGGCAGGGCTC-3’
GAPDH	Forward primer	5’-GAAGGTGAAGGTCGGAGTC-3’
	Reverse primer	5’-GAAGATGGTGATGGGATTTC-3’

### 2.9 Statistical analysis

All statistical analyses and presentations were performed using the R 4.3.0 software package. The differential expression of immune-related genes in LUAD and normal tissues was compared using Wilcox test. Multivariate Cox regression analysis was used to identify immune-related genes associated with poor prognosis in LUAD. T test was used to evaluate the correlation between prognostic genes and transcription factors. Survival analysis was performed using Kaplan-Meier curves. P<0.05, P<0.01 and P<0.001 was considered a statistically significant difference.

## 3 Results

### 3.1 Immune-related genes and enrichment analysis in lung adenocarcinoma was identified

**Figure 1:**
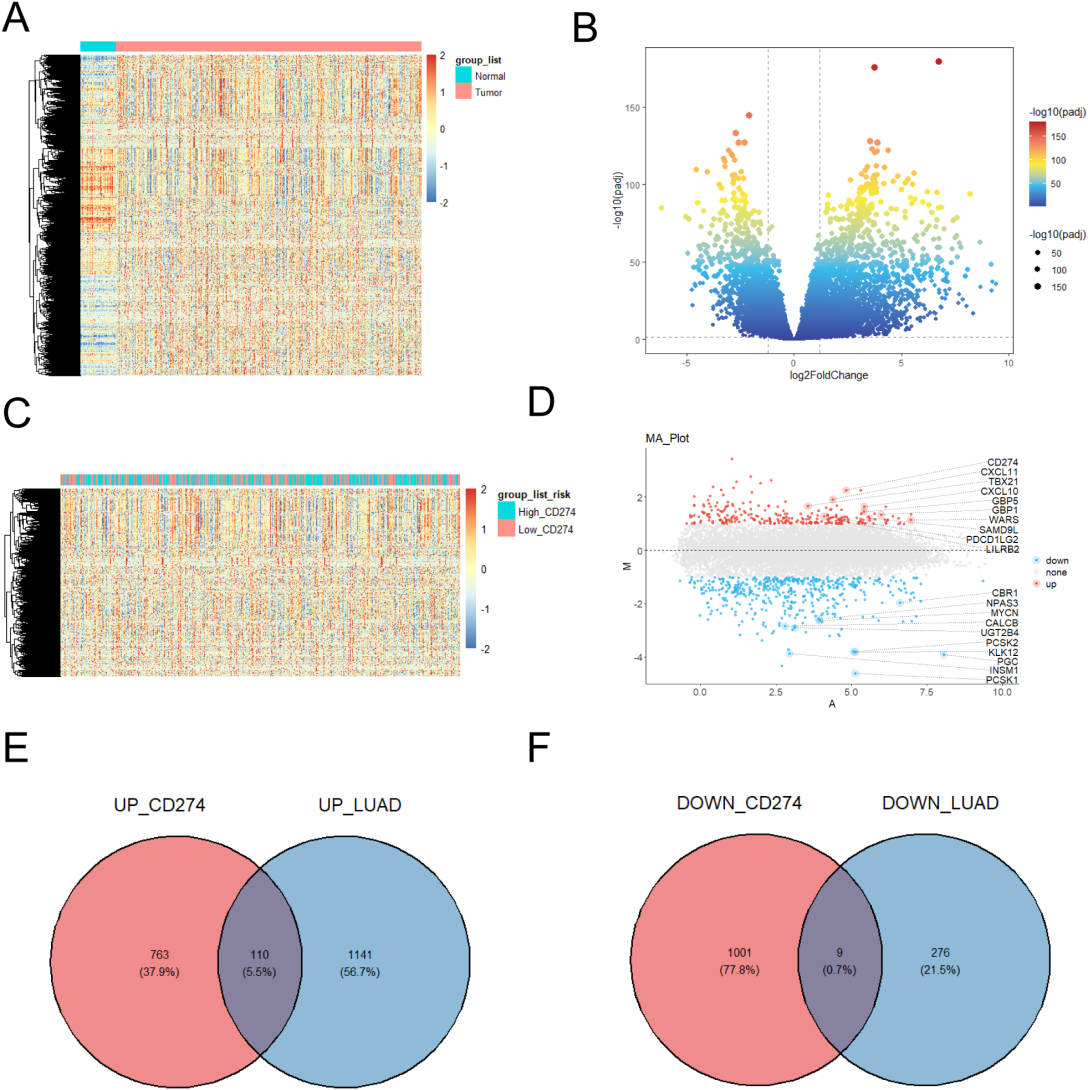
CD274 related-genes were found. **(A)** Tissue adjacent to carcinoma tissue and high expressed genes with lower expression genes heat maps. **(B)** Tissue adjacent to carcinoma tissue and high expression genes with lower expression volcanic figure. **(C)** The amount of gene expression when different CD274 (PD-L1) gene expression of heat map. **(D)** Different gene expression was showed in the volcano. **(E)** High expression of genes and high expressed genes of Wayne figure in lung adenocarcinoma patients. **(F)** Lower expression CD274 the number of patients with lung adenocarcinoma compared with healthy people lower expression of gene Wayne figure.

Analysis of LUAD patient data from the TCGA database identified 1,251 upregulated genes and 285 downregulated genes (**Figures 1A, B**). Using stringent thresholds (adjusted p-value [FDR] < 0.05, |log2 fold change (FC)| > 1) and according to high and low expression of CD274 (PD-L1), we detected DEGs comprising 873 upregulated and 1010 downregulated genes. A heatmap depicting CD274 (PD-L1) expression stratification was shown in **Figure 1C**. Subsequently, Volcano plot demonstrated top 20 differential CD274-related genes (CRGs, **Figure 1D**). Upregulated genes included CD274, CXCL11, TBX21, CXCL10, GBP5, GBP1, WARS, SAMD9L, PDCD1LG2 and LILRB2. Downregulated genes were included CBR1, NPAS3, MYCN, CALCB, UGT2B4, PCSK2, KLK12, PGC, INSM1 and PCSK1. To elucidate the relationship between CD274 and LUAD-associated genes, Venn diagram analysis was performed (**Figures 1E, F**). 873 genes were overlapped with 1,251 LUAD-upregulated genes and the intersected gene was 110. 1,010 CD274-downregulated genes were overlapped with 285 LUAD-downregulated genes and the intersected gene was 9.

### 3.2 Prognostic analysis and survival correlation of immune-related genes was found

**Figure 2:**
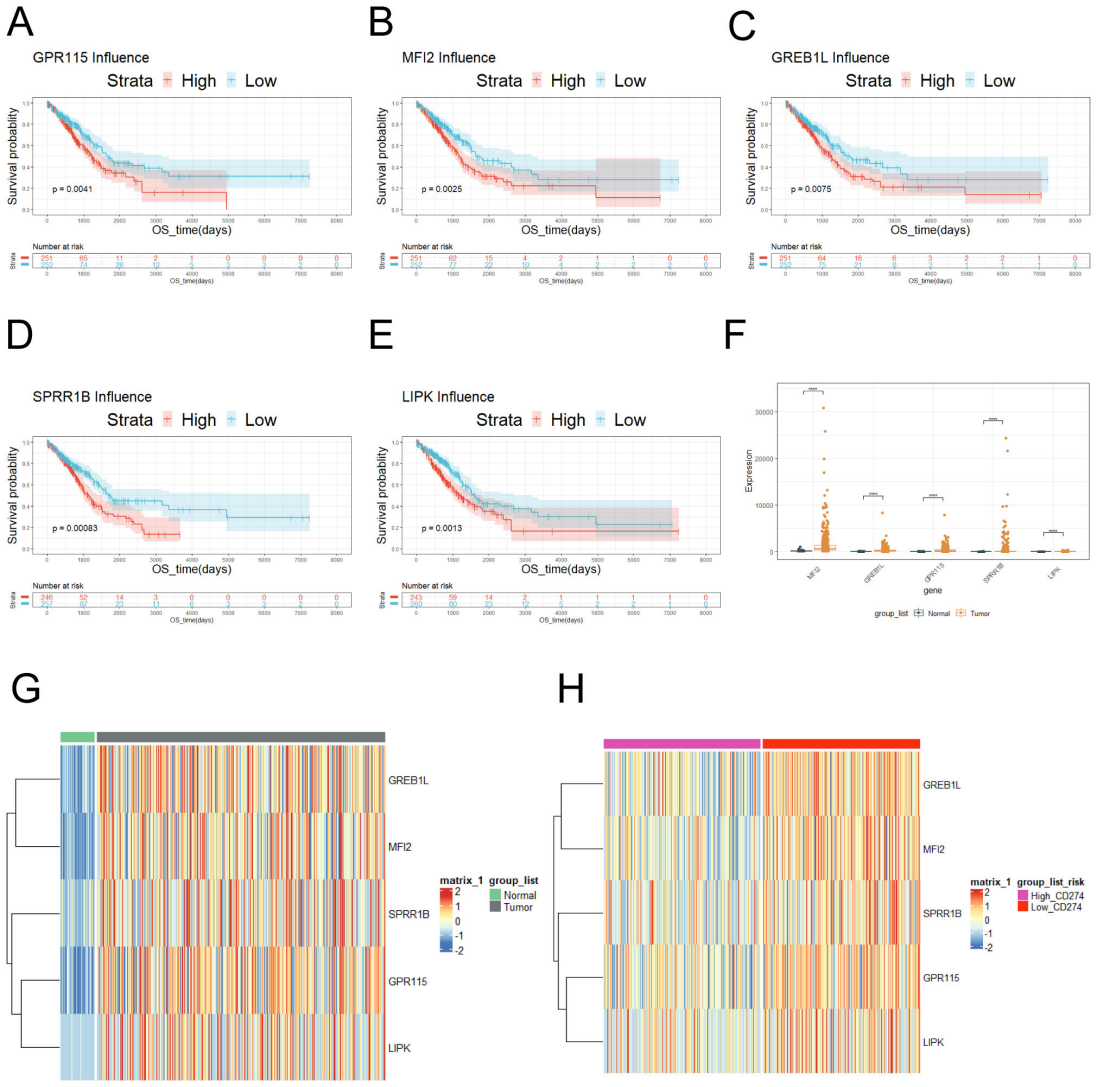
Risk genes associated with prognosis was screened. **(A)** Survival curve of GPR115. **(B)** Survival curve of MFI2. **(C)** Survival curve of GREB1L. **(D)** Survival curve of SPRR1B. **(E)** LIPK survival curve. **(F)** The expression of GPR115, MF12, GREB1L, SPRR1B and LIPK was showed in tumor and normal tissue according to TCGA database. **(G)** The expression of GPR115, MF12, GREB1L, SPRR1B and LIPK was showed on heatmap. **(H)** The expression of GPR115, MF12, GREB1L, SPRR1B and LIPK was showed in tumor tissues according to high and low expression of CD274. ns≥0.05 was considered a non statistically difference. *p<0.05, **p<0.01, ***p<0.001 and ****p <0.0001 was considered a statistically significant difference.

In order to further screen out valid genes, survival analysis was performed to 110 gene and p < 0.01 was screening criteria. Furthermore, Kaplan-Meier survival curves demonstrated that patients with high-expression of GPR115, MF12, GREB1L, SPRR1B, and LIPK exhibited significantly poorer prognosis compared to low-expression (**Figures 2A-F**). What’s more, comparative analysis revealed GPR115, MF12, GREB1L, SPRR1B, and LIPK was upregulated in LUAD samples (**Figures 2G**), which confirmed by hierarchical clustering heatmaps (**Figure 2H**). Intriguingly, heatmaps revealed a progressive increased in expression levels of GPR115, MFI2, GREB1L, SPRR1B, and LIPK in CD274-high expression (**Figure 2H**), suggesting potential co-regulatory mechanisms between these genes and immune checkpoint pathways.

### 3.3 Prognosis-associated genes was screened

**Figure 3:**
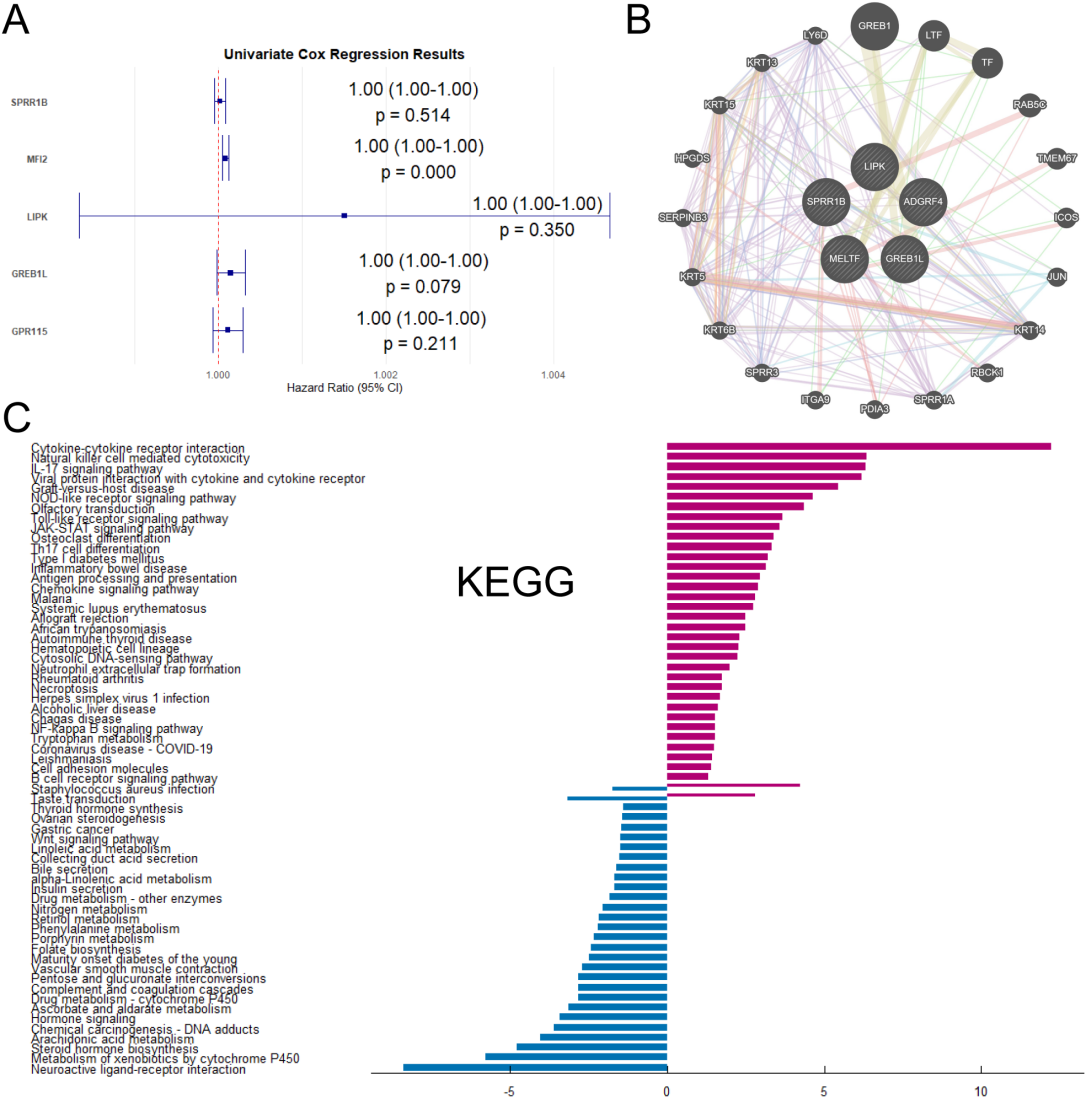
Interactions and enrichment pathways of differential genes between high-CD274 and low-CD274 groups were analyzed. **(A)** Univariate Cox regression analysis of SPRR1B, MFI2, LIPK, GREB1L and GPR115. **(B)** Interaction network analysis of SPRR1B, MFI2, LIPK, GREB1L and GPR115 with other genes. **(C)** KEGG enrichment analysis of DEGs in tumor patients under different CD274 expression levels.

The malignant progression and prognosis of LUAD were related to a variety of genes and factors. Therefore, this section would like to use Univariate Cox regression to demonstrate whether the five genes were associated with the prognosis of LUAD. Univariate Cox regression analysis of SPRR1B, MFI2, LIPK, GREB1L and GPR115 revealed hazard ratios (HR) > 1 (**Figure 3A**), indicating that high expression of these genes was associated with increased mortality risk. Furthermore, to elucidate functional relationships among these genes and their interactome, network analysis was performed (**Figures 3B**), revealing distinct interaction patterns. Co-expression and co-localization of SPRR1B was associated with keratin family genes KRT14, KRT6B and lymphocyte antigen LY6D. MFI2 was physically interacted with endocytic trafficking regulators. GREB1L was shared protein domains with GREB1 (retinoic acid receptor coactivator). Genetic interactions of GREB1L were with transcription factor TF and TMEM67. GPR115 was functionally associations with oncogenic regulators JUN, integrin ITGA9, and epithelial marker KRT5. Finally, we performed KEGG enrichment analysis on DRGs between CD274-high and low expression samples (**Figures 3C**). The results of KEGG analysis showed that the genes with high expression of CD274 in LUAD samples were mostly concentrated in Cytokine-cytokine receptor interaction, IL-17 signaling pathway and other pathways. The genes with low expression of CD274 were concentrated in the pathways of Neuroactive ligand-receptor interaction, Metabolism of xenobiotics by cytochrome P450 and Arachidonic acid metabolism.

### 3.4 Enrichment analysis of differential genes were performed

**Figure 4:**
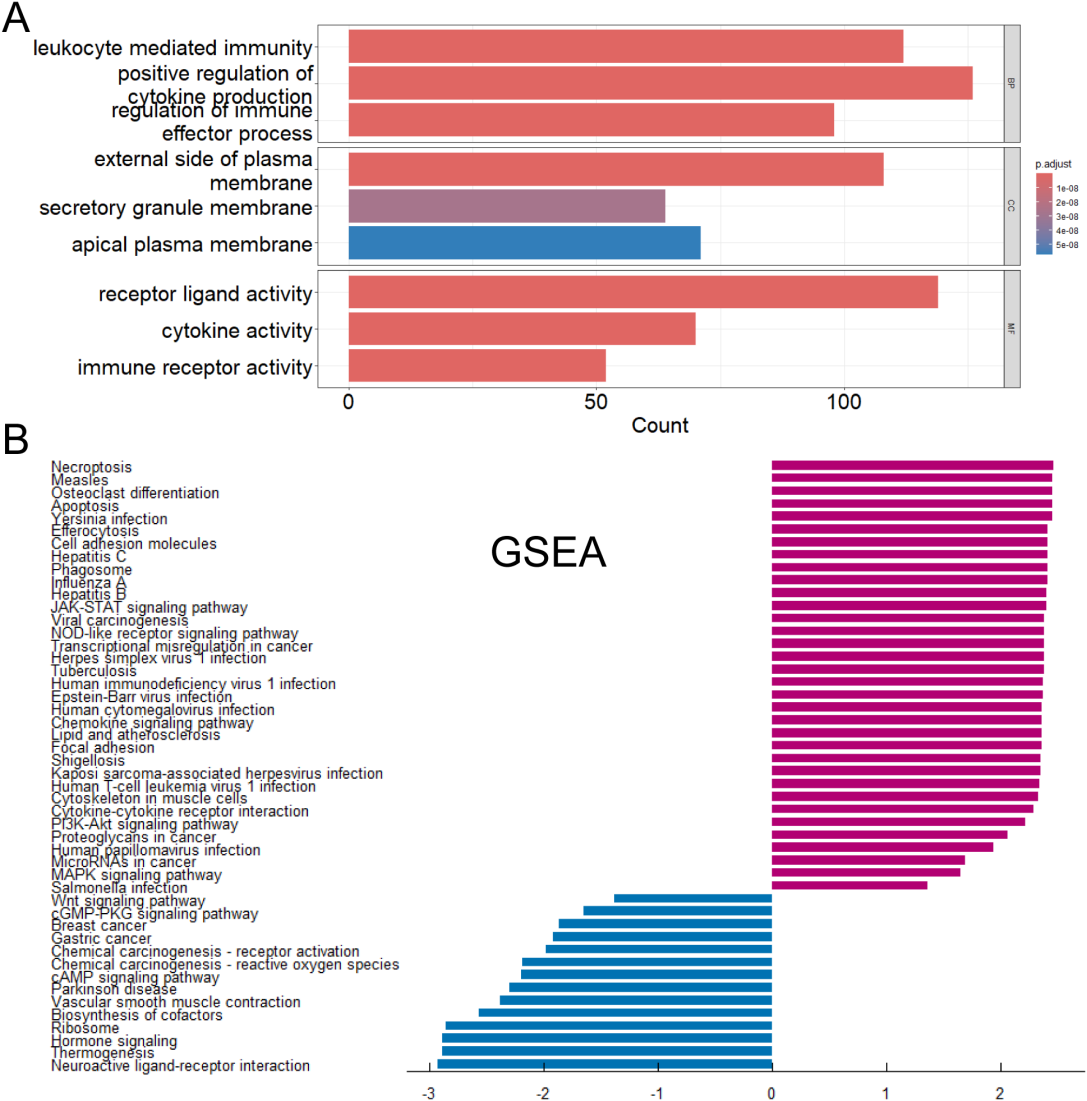
Functions and pathways of DEGs were explored. **(A)** Gene Ontology functional enrichment analysis of differentially expressed genes. **(B)** Gene Set Enrichment Analysis of differentially expressed genes.

We performed GO functional enrichment analysis (**Figures 4A**) and GSEA analysis (**Figures 4B**) to differential genes between CD274-high and low samples. GO analysis showed that most of the differentially expressed genes were mainly enriched in leukocyte-mediated immune response and immune regulation, receptor ligand activity and other processes. These processes played an indispensable role in the occurrence and development of tumors. GSEA analysis showed that the genes with high expression of CD274 and high expression in tumor patients were enriched in Necroptosis, JAK-STAT signaling pathway and Cell adhesion molecules. The genes with low expression of CD274 and low expression in tumor patients were enriched in Neuroactive ligand-receptor interaction, Hormone signaling and cAMP signaling pathway.

### 3.5 The risk scoring model for lung adenocarcinoma with CD274 related genes was constructed

**Figure 5:**
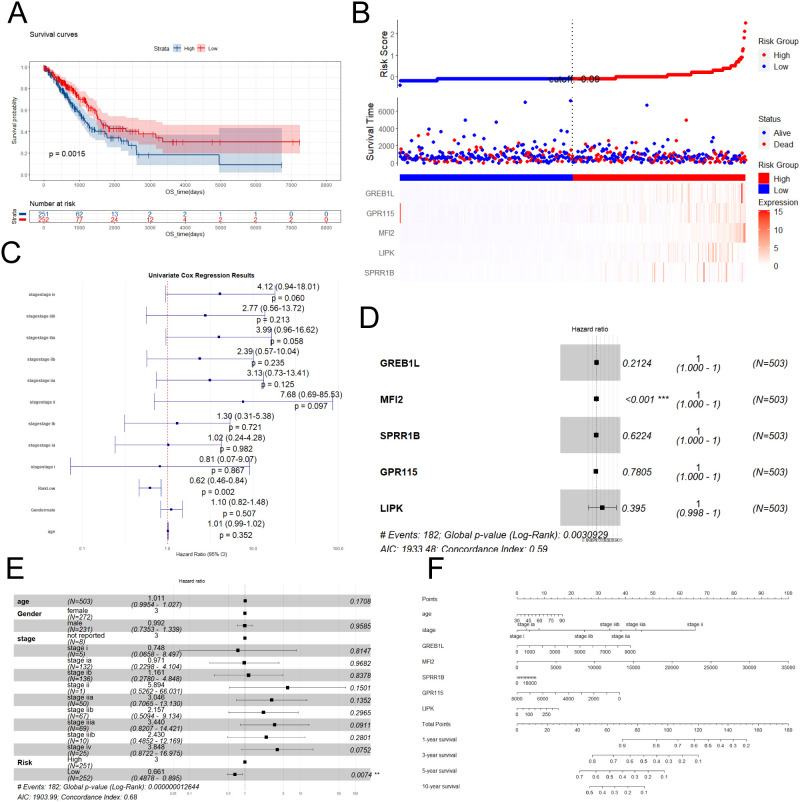
Performant and independent prognostic value of the risk-scoring model was evaluated. **(A)** Survival curves for different risk groups. **(B)** Risk curves, survival status plots and heatmap of modeling gene expression for different groups. **(C)** Univariate Cox regression analysis of various clinical parameters. **(D)** Forest plot of Cox analysis of modeling genes. **(E)** Forest plot of Cox analysis of various clinical parameters. **(F)** Nomogram was performed.

Risk scoring models were a core tool for bridging clinical data and practice by quantifying risk, simplifying decision-making, and facilitating precision medicine. 503 tumor samples were divided into high and low risk groups based on risk scores (**Figures 5A, B**). Survival curves indicated that the high-risk group had a significantly shorter overall survival (OS) than the low-risk group (P = 0.0015). Univariate Cox regression analysis was performed for different influencing factors (**Figure 5C**). Further multivariate Cox regression analysis identified five genes significantly associated with LUAD, which were used to build a prognostic risk scoring model. They were GREB1L (HR = 0.2124, coef = 1.218e - 04), MFI2 (HR < 0.001, coef = 8.705e - 05), SPRR1B (HR = 0.6224, coef = 1.773e - 05), GPR115 (HR = 0.7805, coef = - 3.601e - 05), and LIPK (HR = 0.395, coef = 1.453e - 03). For GREB1L, MFI2, RSPR1B, GPR115 and LIPK, a hazard ratio (HR) > 1 means high expression was linked to high risk (**Figure 5D**). Further multivariate Cox regression analysis was done by combining with other clinical parameters (such as age, gender, and tumor stage) (**Figure 5E**). Results showed that age (HR = 1.011, 95% CI = 0.9954 - 1.027, P = 0.1708), gender (male HR = 3, female HR = 0.992, 95% CI = 0.7353 - 1.339, P = 0.9585), and tumor stage (i stage HR = 0.748, 95% CI = 0.0658 - 8.497, P = 0.8147; ia stage HR = 0.971, 95% CI = 0.2298 - 4.104, P = 0.9682; ib stage HR = 1.161, 95% CI = 0.2780 - 4.848, P = 0.8378; ii stage HR = 5.894, 95% CI = 0.5262 - 66.031, P = 0.1501; iia stage HR = 3.046, 95% CI = 0.7065 - 13.130, P = 0.1352; iib stage HR = 2.157, 95% CI = 0.5094 - 9.134, P = 0.2965; iiia stage HR = 3.440, 95% CI = 0.8207 - 14.421, P = 0.0911; iiib stage HR = 2.430, 95% CI = 0.4852 - 12.169, P = 0.2801; iv stage HR = 3.848, 95% CI = 0.8722 - 16.975, P = 0.0752) were not associated with prognosis in lung adenocarcinoma patients. However, the risk model factor (HR = 0.661, 95% CI = 0.4878 - 0.895, P = 0.0074) was associated with prognosis in lung adenocarcinoma patients. These results confirm the predictive role of the risk score in lung adenocarcinoma prognosis. Importantly, a nomogram was built by combining risk scores with clinical characteristics, with the risk score making a significant contribution to the predictive model (**Figure 5F**).

### 3.6 GO, KEGG and GSEA analysis were performed according to risk scores

**Figure 6:**
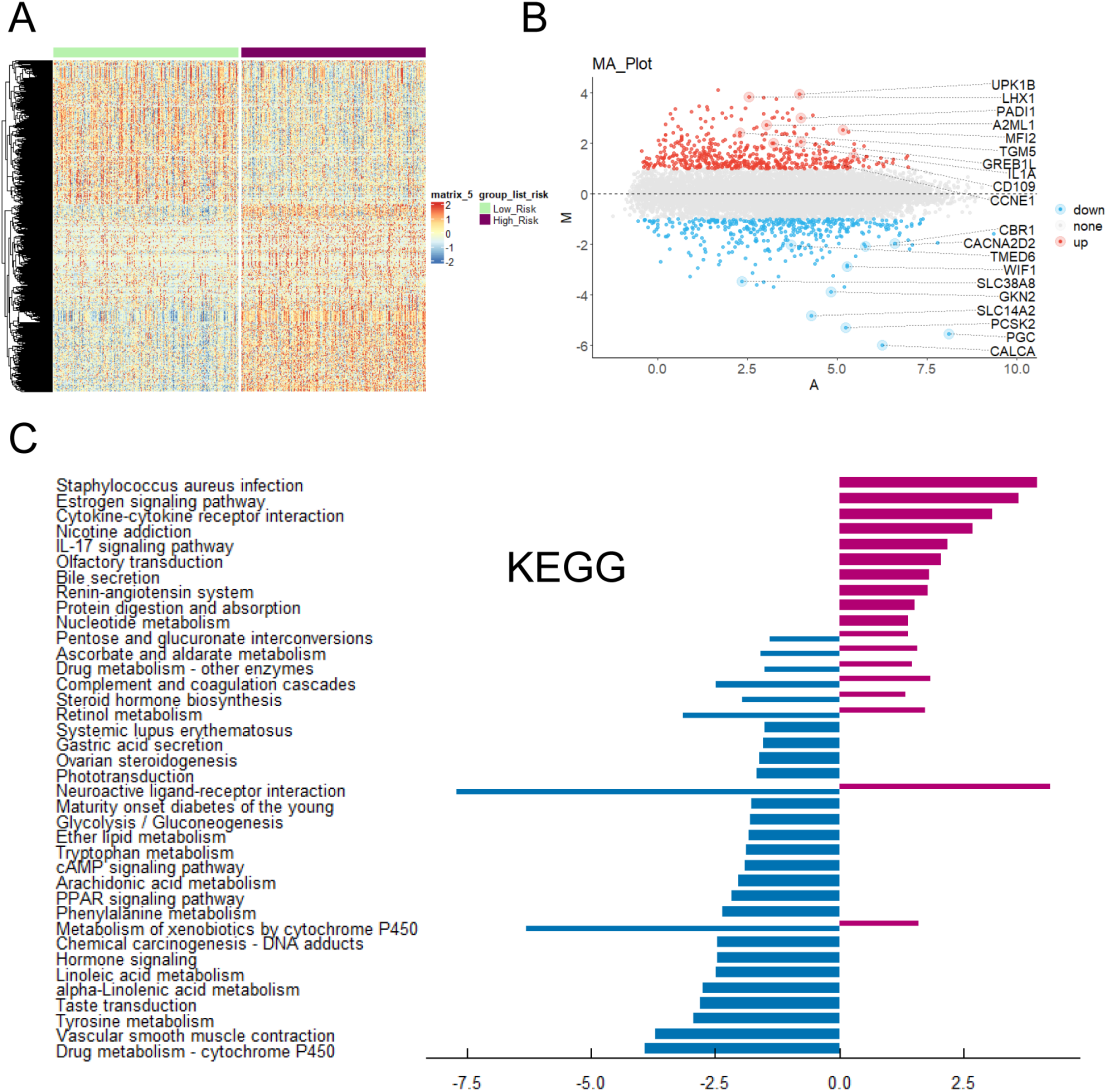
Enrichment analysis of key prognostic genes was conducted. **(A)** Heatmap of DEGs expression and risk scores. **(B)** Volcano plot of the relationship between risk scores and different gene expression levels. **(C)** KEGG enrichment analysis of DEGs in tumor patients under risk scores.

**Figure 7:**
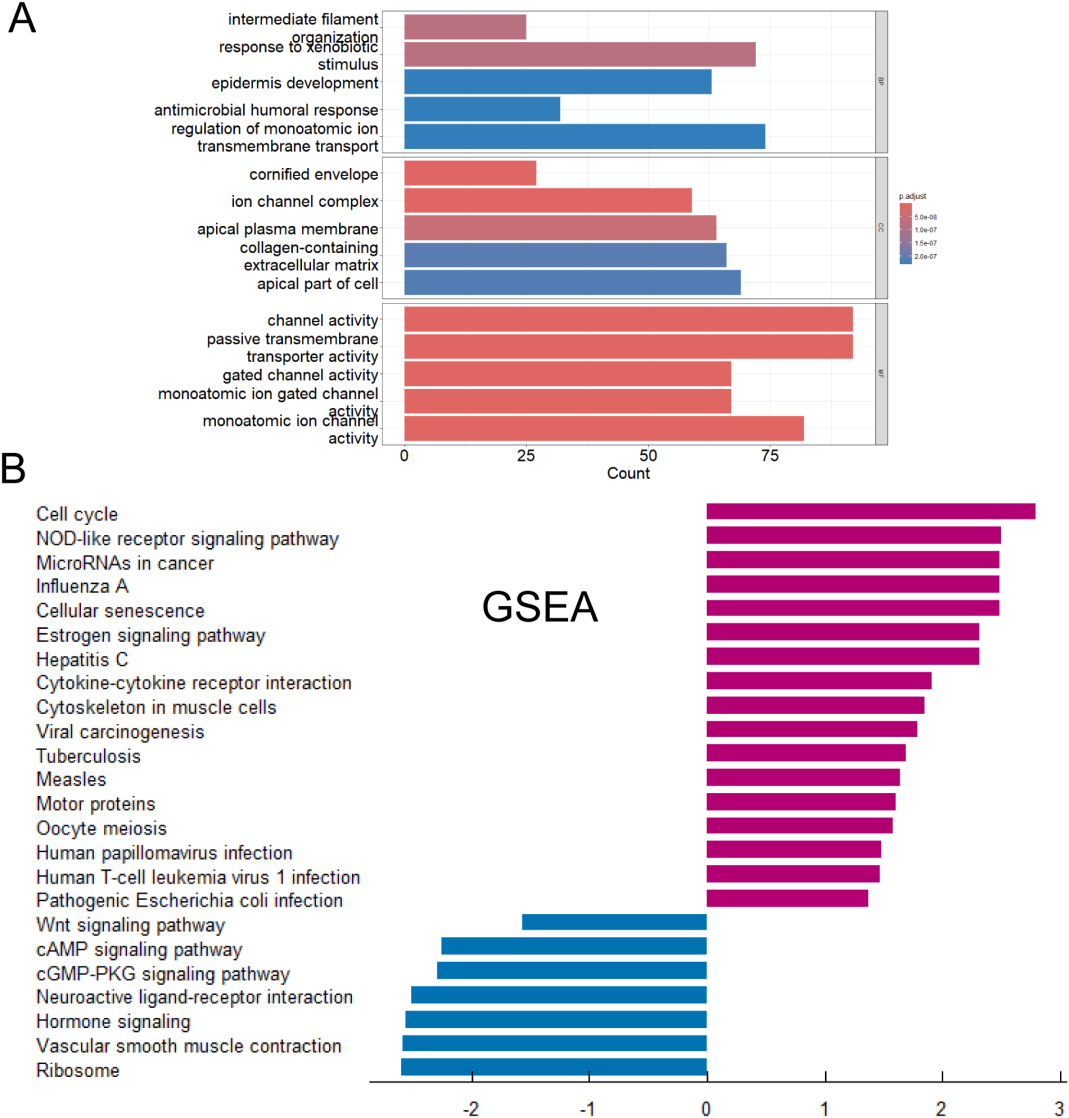
Functions and pathways of key prognostic genes was studied. **(A)** Gene Ontology functional enrichment analysis of associated DEGs. **(B)** GSEA of associated DEGs.

The risk scores of each patient were calculated according to the risk scoring formula. By drawing a heat map (**Figure 6A**), it was found that the risk scores of above five genes (GPR115, MFI2, GREB1L, SPRR1B and LIPK) were high when they were highly expressed, indicating that they were more likely to have poor prognosis when they were highly expressed. Heatmap showed the differential genes between high-risk and low-risk groups (**Figure 6A**). The volcanic map (**Figure 6B**) also showed that top highest genes such as UPK1B, LHX1, GREB1L, PADI1, A2ML1, TGM5, IL1A, CD109, CCNE1 and MFI2 were found. Top lowest genes were CBR1, CACNA2D2, TMED6, WIF1, SLC38A8, GKN2, SLC14A2, PCSK2, PGC and CALCA. We performed KEGG enrichment analysis on DEGs (**Figure 6C**). Staphylococcus aureus infection, Estrogen signaling pathway, Cytokine-cytokine receptor interaction and Phtotransduction so on were involved positively in high-risk groups. Neuroactive ligand-receptor interaction, Metabolism of xenobiotics by cytochrome P450, and Drug metabolism - cytochrome P450 so on were involved negatively in high-risk groups. GO analysis showed these genes were mainly enriched in channel activity, passive transmembrane transporter activity, monoatomic ion channel activity and so on (**Figure 7A**). GSEA indicated they were predominantly involved in cell cycle, NOD-like receptor signaling pathway, cellular senescence and so on (**Figure 7B**).

### 3.7 High risk was associated with immune cell infiltration

**Figure 8:**
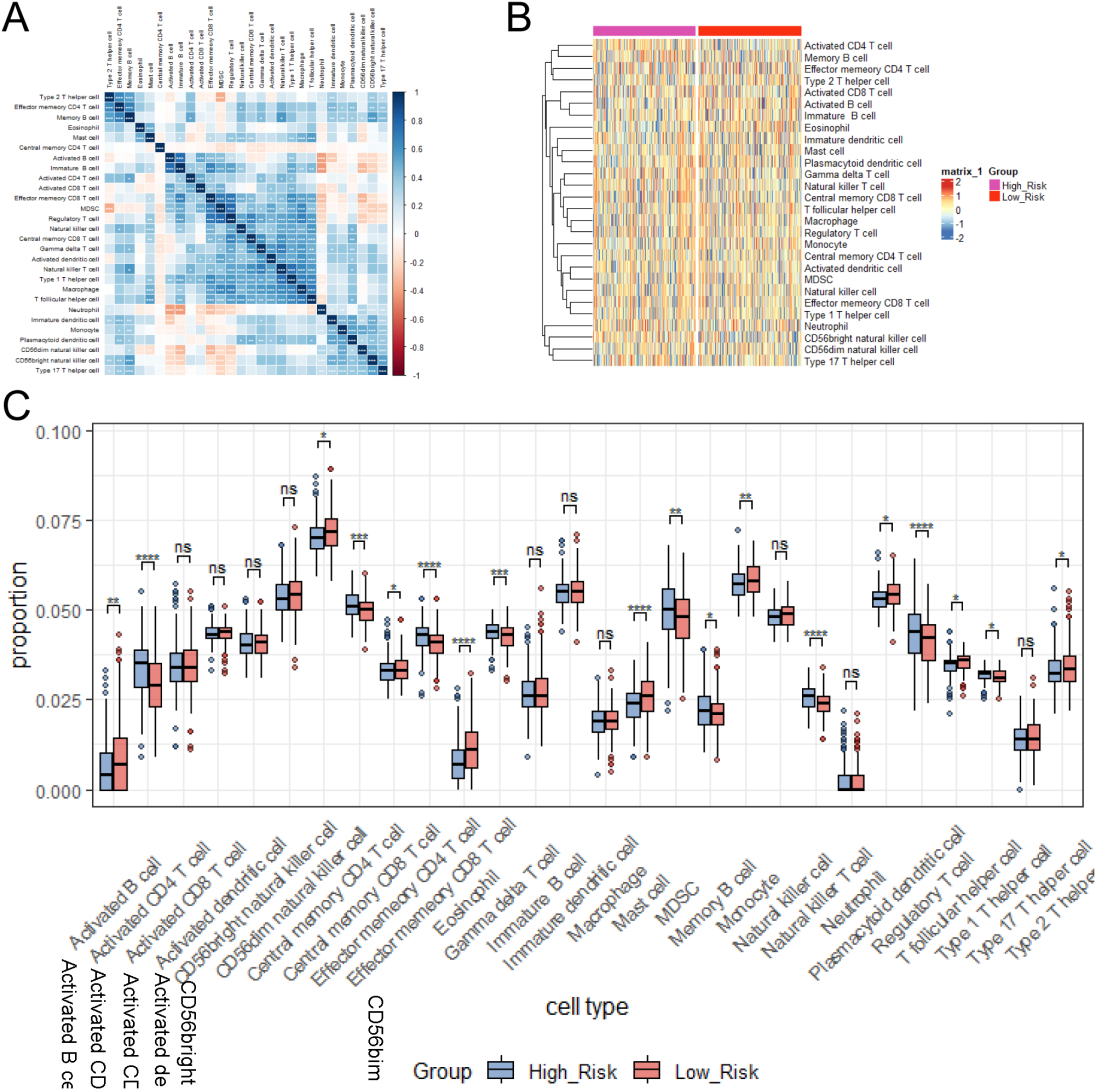
Immune cell correlations and infiltration levels between risk groups were examined. **(A)** Heatmap of immune cell correlation analysis. **(B)** Heatmap of immune cell expression differences between risk groups. **(C)** Relative proportions of immune cell subtypes in risk groups calculated by CIBERSORT algorithm. ns≥0.05 was considered a non statistically difference. *p<0.05, **p<0.01, ***p<0.001 and ****p <0.0001 was considered a statistically significant difference.

Infiltrating immune cells in tumors are able to directly kill tumor cells and are associated with a good prognosis. Natural killer cells (NK cells) induce apoptosis of tumor cells by releasing perforin and granzyme. However, regulatory T cells (Tregs) are able to suppress the immune response and promote tumor immune escape. M2 tumor-associated macrophages secrete proangiogenic factors to support tumor growth and metastasis (43). To explore the correlations among immune cells, we performed pairwise correlation analysis of immune cell gene expression levels and visualized the results with a heatmap using the “ggplot2” package (**Figure 8A**). There were extremely strong correlations between Effector memory CD4 T cells and Type 2 T helper cells, between Effector memory CD8 T cells and MDSCs/regulatory T cells, and between natural killer T cells and Type 1 helper cells. To further evaluate the infiltration levels of immune cells between the high- and low-risk groups, we generated a heatmap (**Figure 8B**). To further assess the infiltration levels of immune cells between high- and low- risk groups, we used the CIBERSORT algorithm to calculate the relative proportions of 28 immune cell subtypes in each lung adenocarcinoma sample. Then, we compared the immune cells with significant differences between the two groups (**Figure 8C**). The high group had higher proportions of Activated CD4 T cells, Effector memory CD8 T cells, Regulatory T cells, and Natural killer T cells, while the low-risk group had higher proportions of Eosinophils and Mast cells.

### 3.8 Optimize the model was validated with data aggregation

**Figure 9:**
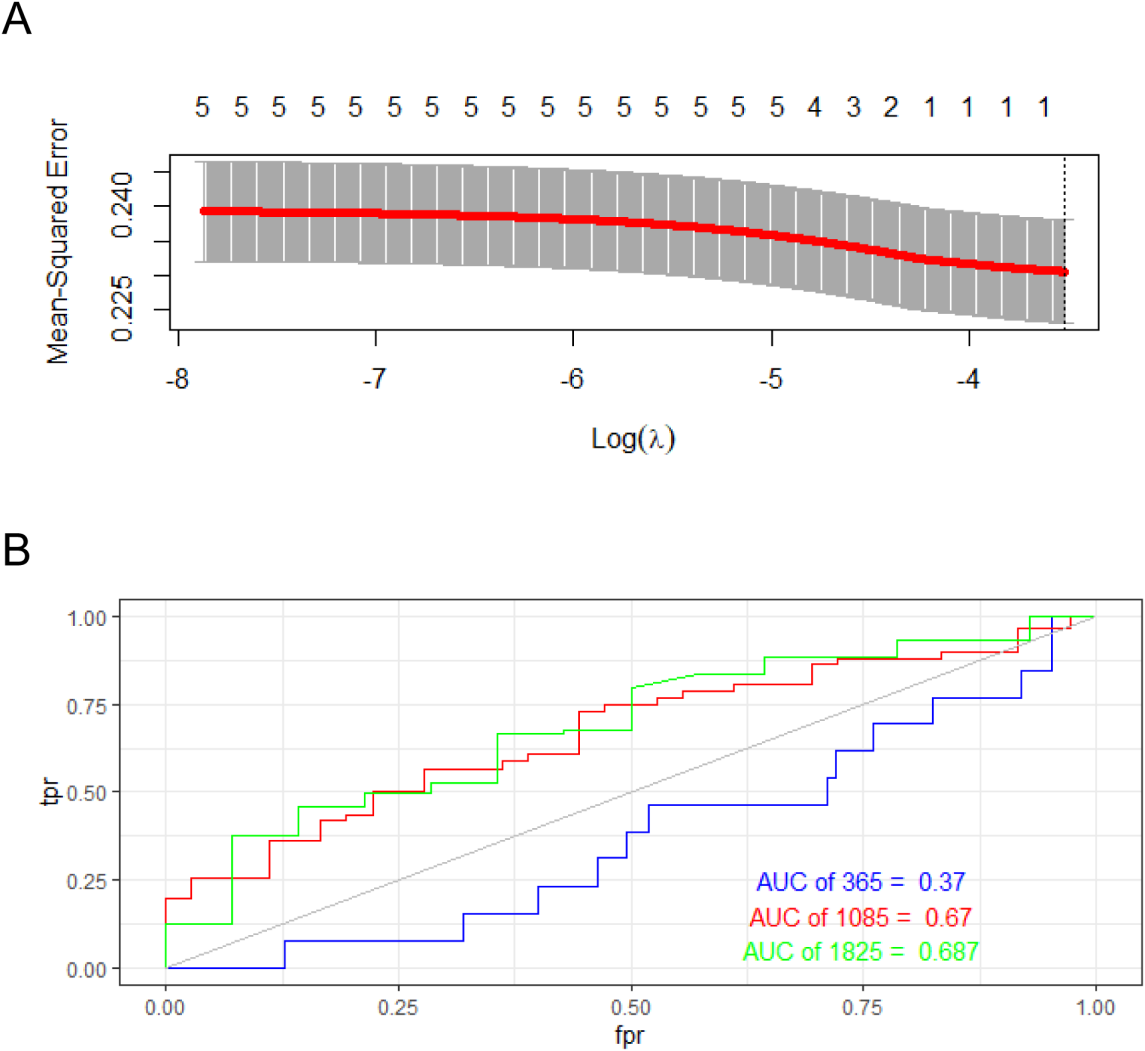
The performance of the prognostic risk-score model was optimized in an internal validation cohort. **(A)** Model optimization by LASSO regression analysis. **(B)** Time-dependent ROC curve.

LASSO regression solves the core problem of high-dimensional data modeling through automatic feature selection and regularization in the prognostic model, making the model more concise, explainable and generalizable. Its combination with survival assays such as Cox LASSO further expands its application value in medical research (44). Therefore, to optimize the model, we used LASSO regression to identify the best genes (**Figure 9A**). With the internal validation cohort, the time-dependent ROC curves revealed 1–3-year AUC values of 0.37-0.687, indicating good predictive performance of the model (**Figure 9B**).

### 3.9 Expression of GREB1L, MFI2, SPRR1B, GPR115 and LIPK was upregulated

**Figure 10:**
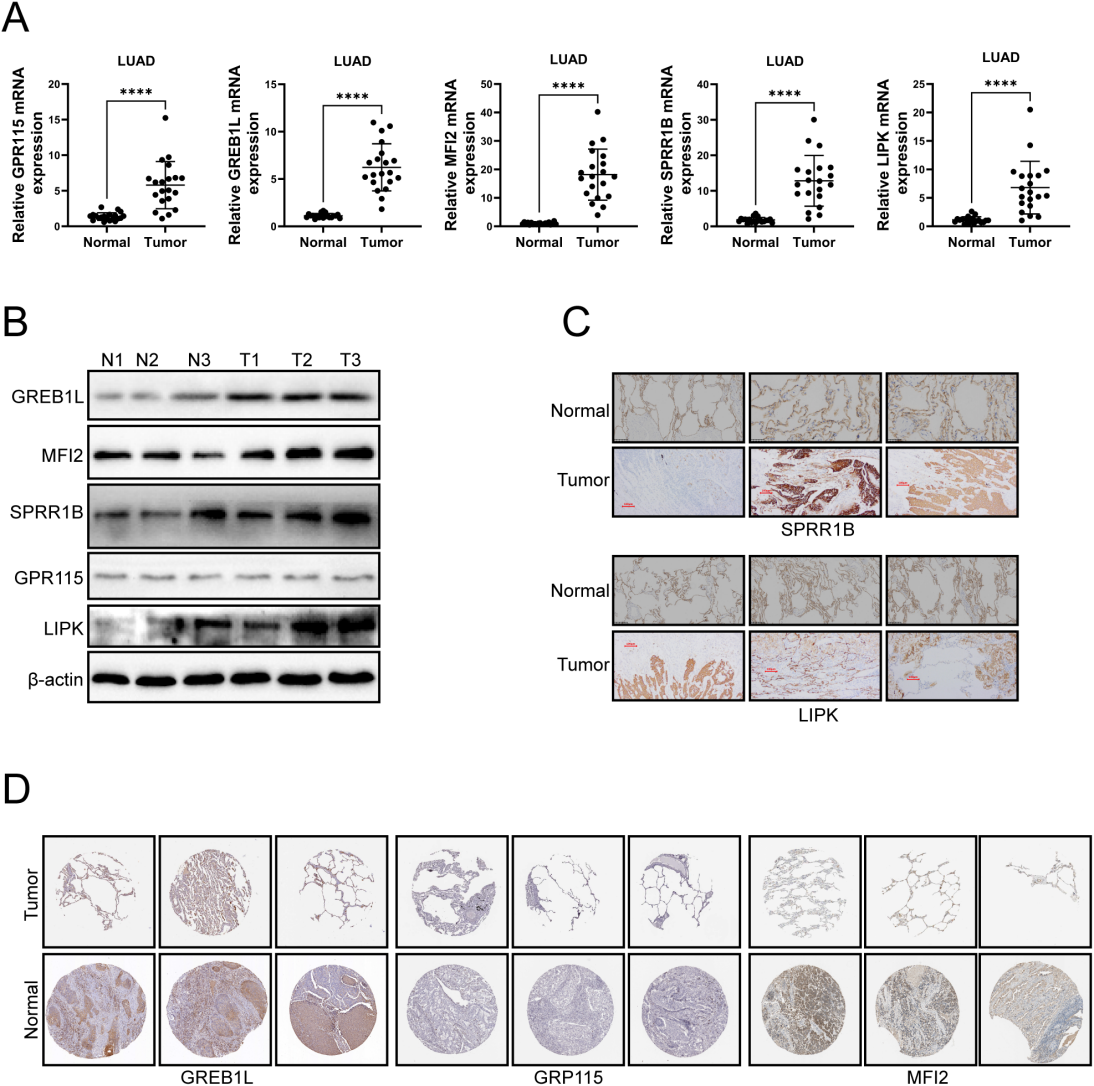
The experimental confirmation of GREB1L, MFI2, SPRR1B, GPR115 and LIPK was performed in LUAD tumor tissues. **(A)** mRNA expression levels of GREB1L, MFI2, SPRR1B, GPR115 and LIPK in LUAD tissues were detected by qRT-PCR. **(B)** Western blotting was used to detect the protein expression levels of GREB1L, MFI2, SPRR1B, GPR115 and LIPK in LUAD tissues. **(C)** Immunohistochemistry was used to detect the protein expression levels of SPRR1B and LIPK in LUAD tissues. **(D)** Protein expression of GREB1L, MFI2 and GPR115 in LUAD samples obtained from the HPA database. ns≥0.05 was considered a non statistically difference. *p<0.05, **p<0.01, ***p<0.001 and ****p <0.0001 was considered a statistically significant difference.

To confirm the expression of PD-L1 related genes, PCR, Western blotting and immunohistochemistry were used to verify the expression levels of GREB1L, MFI2, SPRR1B, GPR115 and LIPK in LUAD tumor tissues. PCR results showed elevated expression of GREB1L, MFI2, SPRR1B, GPR115 and LIPK mRNA in LUAD tumor tissues (**Figure 10A**). Western blotting results showed that the protein expression levels of GREB1L, MFI2, SPRR1B and LIPK were increased, but the expression levels of GPR115 did not change significantly (**Figure 10B**). Immunohistochemistry confirmed that the expression levels of GREB1L, MFI2, SPRR1B, and LIPK proteins were elevated in LUAD tissues, and GPR115 did not change significantly (**Figure 10C, D**). These results suggest that GREB1L, MFI2, SPRR1B, GPR115 and LIPK might be involved in the pathological process of LUAD.

## 4 Discussion

As the main subtype of NSCLC, lung adenocarcinoma is highly heterogeneous and has a complex immune microenvironment, which makes prognosis prediction and treatment strategy formulation a great challenge (4, 5). This study provides a new perspective for the precise typing of lung adenocarcinoma and the exploration of immunotherapeutic targets by integrating multi-omics data, systematically screening key genes related to immune regulation and prognosis, constructing a risk scoring model, and revealing their association with immune microenvironment characteristics.

As the core molecule of the PD-1/PD-L1 immune checkpoint pathway, the expression level of CD274 (PD-L1) directly affects the immune escape ability of tumors (45, 46). In this study, it was found that the CD274 high expression group had significant overlap with the highly expressed genes in lung adenocarcinoma patients (110 genes), and were enriched in cytokine-cytokine receptor interaction and IL-17 signaling pathway. This is consistent with previous studies in which IL-17 promotes PD-L1 expression in tumor cells by activating STAT3 signaling and recruiting both myeloid suppressor cells (MDSCs) and regulatory T cells (Tregs) to form an immunosuppressive microenvironment (47, 48). It is worth noting that there were only 9 intersections between the CD274-low expression group and the low-expression genes of lung adenocarcinoma, suggesting that the high expression of CD274 may be the dominant phenotype of immune escape in lung adenocarcinoma. This finding supports the potential of PD-L1 inhibitors in the treatment of lung adenocarcinoma, especially in the patient population with high CD274 expression.

In addition, CD274 genes associated with high expression are enriched in Necroptosis and Cell adhesion molecules, suggesting that CD274 may influence anti-tumor immune responses by regulating tumor cell death patterns (such as immunogenic death) or cell to cell interactions (49–51). However, the direct association between CD274 expression and prognosis needs to be further verified, as it may be regulated by molecular characteristics such as tumor mutation load (TMB) or microsatellite instability (MSI).

Five high-risk genes selected in this study (GPR115, MF12, GREB1L, SPRR1B, LIPK) showed independent prognostic value in univariate and multivariate COX analyses (HR>1), and the constructed risk scoring model showed some generalization ability in external validation. Functional annotation and interaction network analysis of these genes reveal their underlying biological mechanisms. SPRR1B is co-expressed with keratin genes (KRT14, KRT6B), which may promote tumor invasion by regulating EMT. GREB1L, as a co-activator of estrogen receptors, may drive tumor proliferation through hormone signaling pathways. LIPK is involved in lipid metabolic reprogramming and may affect immune cell infiltration by altering cell membrane fluidity. MFI2 may enhance the migration and invasion ability of tumor cells through integrin-extracellular matrix interactions, or affect the drug resistance and immune escape of tumor cells by regulating iron-dependent metabolic pathways (such as lipid peroxidation). GPR115 is abnormally expressed in a variety of malignant tumors (such as breast cancer and glioma), which may promote tumor invasion and drug resistance by activating MAPK/ERK or PI3K/AKT pathways (28–31). In lung adenocarcinoma, the expression of GPR115 was significantly up-regulated, and was significantly associated with TNM stage progression, distant metastasis, and shortened overall survival. The enrichment pathways of these genes (e.g., Staphylococcus aureus infection, light signaling) had not been adequately studied in lung adenocarcinoma, suggesting that they might indirectly influence tumor progression through regulation of the microbiome or environmental stress response. Importantly, the high expression of GPR115, MF12, GREB1L, SPRR1B, LIPK were verified by *in vitro* and *vivo* experiments.

By analyzing the immune cell infiltration pattern using CIBERSORT algorithm, this study found that the high-risk group was characterized by increased infiltration of activated CD4+ T cells, effector memory CD8+ T cells, and Treg cellswhile the low-risk group was dominated by eosinophils and mast cells. This phenomenon might reflect two distinct immune states.

Despite the increased number of effector T cells, their function might be suppressed by Treg cells and MDSC, resulting in an “immune depletion” phenotype. This hypothesis was supported by pathway enrichment results. that High risk genes were enriched in cell cycle and NOD-like receptor signaling pathways, suggesting that sustained inflammatory responses minght accelerate genomic instability while activating immune checkpoint molecules (such as PD-L1).

Eosinophils and mast cells might inhibit Th1 anti-tumor immunity by releasing Th2 cytokines such as IL-4 and IL-13, but their metabolic pathways (such as cytochrome P450) that were highly expressed at the same time might play a protective role by clearing carcinogens (52, 53). This finding highlighted the complexity of the immune microenvironment. The increase in the number of immune cells alone might not represent anti-tumor activity, and comprehensive analysis should be combined with functional status and spatial distribution.

The innovation of this study was reflected in the following aspects. For the first time, GPR115, MF12 and other genes were incorporated into the prognosis model of lung adenocarcinoma, and their predictive value independent of traditional clinical stages provided a new tool for individualized treatment. By integrating KEGG metabolic pathway and immune cell infiltration data, the hypothesis that metabolic reprogramming may affect prognosis by regulating immune cell function was proposed. The constructed column graph visually showed the relationship between risk scores and survival probability, which was convenient for clinicians to quickly evaluate patient prognosis.

The expression of PD-L1 (54), tumor mutational burden (TMB) (55), specific gene mutations, tumor-infiltrating lymphocytes (TIL) (56), antigen presentation defects (57), and gene expression profiles (GEPs) (58) could be used as predictive biomarkers of immunotherapy efficacy. Among them, PD-L1 expression, TMB (≥10 mut/Mb), microsatellite instability (MSI-H), and mismatch repair deficiency (MMR) had been approved by health regulatory agencies as predictive biomarkers for immunotherapy in patients with NSCLC (55, 59–62). PD-L1 and TMB, as composite biomarkers, had stronger predictive power than their use alone (55, 63). Recent studies had shown that mutations in TP53, one of the tumor suppressor genes, were associated with high PD-L1 expression and TMB (64). In addition, in various cancers, patients with TET1 mutations had longer progression-free survival (PFS) and overall survival (OS) after receiving immunotherapy than patients without TET1 mutations (65). Gene mutations might be associated with the DNA damage and repair (DDR) pathway, which improved tumor immunogenicity by accumulating pseudo-DNA damage responses, and had shown good efficacy in immunotherapy (66, 67). These studies focused on PD-L1 as a predictive biomarker to predict the efficacy of immunotherapy, however, there was a lack of PD-L1-related predictive biomarker studies. Therefore, this study focused on exploring whether PD-L1-related predictive biomarkers had a predictive effect on the prognosis of patients with LUAD. It is found that GREB1L, MFI2, SPRR1B, GPR115 and LIPK were PD-L1-related genes that predicted the prognosis of LUAD patients. Our study could help predict the prognosis of LUAD patients with high PD-L1 expression.

However, the study has the following limitations. The function of key genes (such as SPRR1B) had not been verified by experiments, and whether it was directly involved in immune regulation was still unclear. The AUC value of verification was low (0.687), which might be due to sample heterogeneity or batch effect, and the feature selection needs to be optimized by multicenter queue or deep learning algorithm.

In conclusion, the PD-L1 risk prediction model in this study could effectively predict the prognosis of patients. The construction of PD-L1 risk model was of great significance for the treatment of lung adenocarcinoma.

## Data Availability

The original contributions presented in the study are included in the article/supplementary material. Further inquiries can be directed to the corresponding authors.
